# Impact of advanced practice nurses in hospital units on compliance with clinical practice guidelines: a quasi-experimental study

**DOI:** 10.1186/s12912-022-01110-x

**Published:** 2022-11-29

**Authors:** Sandra Pol-Castañeda, Miguel Angel Rodriguez-Calero, Carlos Javier Villafáfila-Gomila, Ian Blanco-Mavillard, Concepción Zaforteza-Lallemand, Francisco Ferrer-Cruz, Joan Ernest De Pedro-Gómez

**Affiliations:** 1 Hospital Son Llàtzer, 07198 Palma, Balearic Islands Spain; 2 Care, Chronicity and Health Evidences (CuRES) Research Group, Health Research Institute of the Balearic Islands (IdISBa), 07010 Palma, Balearic Islands Spain; 3 Balearic Islands Health Services, 07003 Palma, Balearic Islands Spain; 4 Hospital Manacor, 07500 Manacor, Balearic Islands Spain; 5 Hospital Comarcal d’Inca, 07300 Inca, Balearic Islands Spain; 6 Department of Nursing and Physiotherapy, University of the Balearic Islands, 07122 Palma, Balearic Islands Spain

**Keywords:** Evidence-based practice, Advanced practice nursing, Nurses, Practice guideline, Pressure ulcer, Vascular access devices, Implementation science

## Abstract

**Background:**

Incorporating the best available evidence into clinical practice is a determining challenge for healthcare professionals and organisations. The role of advanced practice nurses is viewed as a facilitator to adapt guideline recommendations to suit specific contexts and to overcome barriers to implementation. In this study, we evaluate the impact of advanced practice nurses on clinical indicators of hospitalised patients and on adherence to recommendations derived from two clinical practice guidelines (pressure ulcer prevention and treatment and vascular access device management).

**Methods:**

Quasi-experimental study in five intervention (IU) and five control (CU) hospital units at three hospitals in Spain (period 2018–19). Five advanced practice nurses were incorporated into IU, with the intention that would produce attitudinal changes and enhance the skills and knowledge of the nursing team regarding 18 clinical practice recommendations. In this study, 41 indicators were evaluated through direct observation of all patients admitted, at monthly intervals for 1 year. Outcomes were assessed by means of a descriptive, multi-line regression and association analysis.

**Results:**

The study population was composed of 3742 inpatients admitted for pressure ulcer assessment and 2631 fitted with vascular access devices. By the end of the study period, all variables had improved in the IU, where average compliance with recommendations was statistically significantly higher (pressure ulcer guidance 7.9 ± 1.9 vs 6.0 ± 1.7. OR 1.86, 95% CI 1.67–2.05; vascular access devices guidance 5.4 ± 1.4 vs 4.4 ± 1,6. OR 1.06, 95% CI 0.95–1.17). The prevalence of pressure lesions and catheter-related adverse events decreased statistically significantly in the IU compared to the CU. The prevalence of pressure ulcers decreases (5.7% in IU vs 8.7% in CU *p* < 0.005) as well as the prevalence of adverse events related to the catheter (14% In IU vs 21.6% in CU *p* < 0.005). The unnecessary catheters decressed in IU 10.9% VS CU 15.8% (p < 0.005).

**Conclusions:**

The incorporation of an advanced practice nurse statistically significantly improves clinical indicators related to the prevention and treatment of pressure ulcers and to the management of vascular access devices.

**Trial registration:**

ISRCTN18259923 retrospectively registered on 11/02/2022.

**Supplementary Information:**

The online version contains supplementary material available at 10.1186/s12912-022-01110-x.

## Background

Incorporating the best available evidence into nurses’ clinical practice continues to be a challenge for healthcare professionals and organisations. Shortcomings in this respect result in inconsistencies between the recommendations made in clinical practice guidelines (CPGs) and the reality of the care offered [1, 2]. Over the last 30 years, studies of this situation have identified various contributory factors, some corresponding to the organisational culture (such as lack of team support, inadequate resources, poor leadership, insufficient communication with other disciplines), while others concern the nurses’ own characteristics (competence, attitudes, perceptions, skills, motivation, resistance to change, etc.) or are intrinsic to the evidence itself [3–6]. The joint impact of these factors can produce considerable variability in clinical practice, as decision-making is often based purely on the experience and judgment of the healthcare personnel concerned [7–9].

Previous efforts to create CPGs to enhance and standardise professional practice have mostly failed because they were not accompanied by concrete implementation plans or were not based on solid theories and robust methods. These deficiencies provoked delays in implementation and consolidated suboptimal healthcare and variability in clinical practice [10–16].

In this context, various research studies and models of the implementation of research findings recommend the use of CPGs as a means of enhancing health care and outcomes [17–19]. In Spain, the incorporation of Advanced Practice Nurses (APNs) into the health system is viewed as a useful response to the need to improve the application of CPGs. The use of different nursing leadership roles with the ability to influence their environment is a highly studied strategy [3, 18, 20–22], but in addition, the close and daily contact of the APNs with the nursing team, maintaining their own patient care activity as a benchmark in care, places them in an optimal position, making it possible to adapt the CPGs recommendations to suit specific circumstances and to overcome barriers to implementation [23]. APNs provide support in problem-solving, via individual and collective actions, and usually receive valuable support from the health system [20, 24].

Previous studies have explored the role of APNs as agents of change and as facilitators of the implementation of evidence [13, 24–26]. We propose that this role be formally incorporated into hospitalisation units, under the denomination of Advanced Practice Hospitalisation Nurses (APHNs). The integrated Promoting Action on Research Implementation in Health Services framework [27, 28] was used as a guide to identifying the necessary elements to design and execute the implementation study, where the APHN functioned as the main active element that embodied the role of facilitator. The Theory of Planned Behaviour [29] was used to understand the determinants of the registered nurses’ clinical behavior change process, thus guiding the APHN’ training and helping them to identify possible implementation strategies. Taken in combination, these theoretical approaches provide a sound framework for establishing facilitation as a crucial forerunner of effective implementation [25, 26, 30]. The previously-published protocol for the present study described the interventions carried out, with respect to each of the variables considered [31], taking due account of the context and the organisational climate in which improvements were sought [32, 33] and the consequent improvements in clinical outcomes [34].

Although nurses constitute the most numerous element of human capital in the health system, relatively little research attention has focused on the roles they play and the value added to clinical outcomes [35]. In the present study, our aim is to determine the added value provided by APHNs. In this respect, few indicators have been published to help establish the quality of care [36], and so our analysis is based on indicators extracted from two CPGs published by the Spanish National Health System. These guidelines refer to the treatment and prevention of pressure ulcers and the management of vascular access devices [37, 38]. In most adult hospitalisation units, both pressure ulcers and vascular access are important aspects of primary and transversal nursing care and are closely related to the quality of the process and to the resulting morbidity and mortality [39, 40].

This study considers, for the first time with respect to the Spanish Health Service, the value of incorporating APHNs into hospital units as a facilitation strategy to improve the implementation of research-based evidence, and responds to the request from the international arena to countries that are recently embraced advanced practice roles, as is the case of Spain, to share evidence of their own experiences to ensure a sustainable transition [41].

## Methods

### Aim

The main objective of this study is to determine the impact of the incorporation of APHNs on the clinical indicators of hospitalised patients and on the level of adherence of the nursing team to the recommendations derived from two CPGs, and thus the implementation of research-based evidence.

### Design

In this quasi-experimental study, the clinical indicators derived from two CPGs currently in use in the Balearic Islands Health System were evaluated by monthly on-site audits [37, 38].

This study is part of a mixed method project developed to explore the outcomes obtained when APHNs are incorporated into conventional hospital units. The protocol for this project has been published previously [31]. In the present paper, we report the findings of the first phase of the study, in which clinical indicators were monitored and a quantitative methodology was applied (trial registry ISRCTN18259923; Registration date 11/02/2022).

The study was carried out at three public hospitals (a university hospital and two general hospitals) in the Balearic Islands (Spain). For the purposes of this analysis, ten medical and surgical units were selected, with 5 units as the intervention group, and the remaining five as the control.

Before selecting these units, the organisational climate was characterised, using the validated Practice Environment Scale-Nursing Work Index questionnaire [42]. This instrument measures the degree to which a certain environment is favourable for the development of a recommended nursing practice. At each participating hospital, intervention and control units with a similar patient profile and comparable total Practice Environment Scale-Nursing Work Index score (± 5%) were selected (see Additional file 1).

The intervention consisted of the incorporation of an APHN within each unit involved, to participate in activities appropriate to the context in question, with specific interest in providing support to health teams, motivating attitudinal change regarding skills, abilities and knowledge and seeking to ensure the implementation of CPGs recommendations and the avoidance of low-value practices. Among other actions, the APHNs extended awareness of the project, assisted in the implementation of the CPGs, worked with the team to establish objectives, provided training for the health team, contributed to the planning of changes in routines, materials, techniques, etc., evaluated the results obtained, provided periodic feedback to the health team, adjusted the interventions as necessary, and offered support and mentoring.

The APHNs were selected from the registered nurses deployed within the intervention units at the start of the study. This selection was based on each individual’s leadership qualities and on the score obtained in the Advanced Practice Nursing Competency Assessment Instrument [43]. A second nurse from the team (the ‘support RN’) was also selected to stand in for the primary APHN if necessary. All involved – APHNs, support RNs and ward supervisors – took part in an ad hoc training programme to develop the competencies associated with advanced practice and with the CPGs being evaluated. Further details of this programme are available in the research protocol [31]. These personnel were also instructed in the required methods of data collection and recording. Monthly meetings were held to monitor the intervention, in which the APHN and other team members participated, and individual contacts were also available if needed.

In the units forming the control group, no type of intervention was carried out, although the personnel did receive general information about the project before it started, through the unit supervisors. Nevertheless, these units were given the normal CPGs, as recommended by the health system.

### Sample/participants

The sample was composed of all adult patients hospitalised in the intervention and control units on the specific days in which audits were carried out, 1 day per month for each CPG. Patients in terminal care were excluded.

To calculate the minimum sample size, a pressure ulcer incidence of 8.6% and a catheter-associated adverse event incidence of 41% were assumed. These values were obtained from observational studies carried out locally [44, 45], in which the interventions performed reduced the incidence of these events by 10%. To detect this difference between the two proportions as statistically significant, accepting an alpha risk of 0.05 and a beta risk of 0.2 in a bilateral test, we calculated that a minimum sample size of 476 patients would be needed for the pressure ulcers guideline (238 in each group) and 722 for the vascular access guideline (361 in each group). These values were obtained using the ARCSIN approximation. A 12-month follow-up period was established for each unit to ensure the necessary sample size was obtained and to detect any seasonal variations.

### Data collection

In both control and intervention groups, sociodemographic variables (age, sex, date, unit of admission) were recorded, as well as process and outcome variables derived from the two CPGs (see Additional file 2). In the case of the pressure ulcer assessment, after collecting the sociodemographic variables, the APHN evaluated the patient’s risk of developing a pressure ulcer according to the Braden scale and evaluated the presence of pressure ulcers. A patient at risk of pressure ulcer was defined as a Braden score of < 17 points in patients younger than 75 years or < 19 points in patients older than 75 years. A patient at risk or currently presenting pressure ulcers was considered a “candidate for extended care” and only then, the rest of the variables were collected (see Additional file 2). The variables considered as potential effect modifiers were the time in which the audit was carried out (“time of the audit”, measured in months) and unit of admission (APHN vs control). In the case of pressure ulcer guideline, the variable “risk of pressure ulcer” was also included.

Data collection began simultaneously with the onset of APHN activity in the unit (baseline), by direct observation of hospitalised patients on a predefined day, at one-month intervals, for 12 months. Independent audits were conducted to assess issues related to the prevention and care of pressure ulcers, on the one hand, and the insertion and maintenance of peripheral vascular catheters, on the other. The data collection period was from April 2018 to September 2019.

The audits were carried out by registered nurses specifically trained for this task to ensure data homogeneity. The data were stored in an anonymised database for later analysis.

## Data analysis

A descriptive analysis was made of all the study variables in order to define the characteristics of the study group, using frequencies and percentages for the qualitative variables and means and standard deviations for the quantitative ones. The Kolmogorov-Smirnov test was used for the analysis of normality. The findings for each process and outcome variable were evaluated by comparing each month of the study, together with the data accumulated by the end of the study period. For the variables regarding adherence to the CPG recommendations, the differences between the baseline and the last month of the study were evaluated, comparing the degree of compliance between groups, and the changes observed within each group. To evaluate inter-group differences, Student’s t-test was used for the quantitative variables and the chi-square test or Fisher’s exact test for the qualitative ones.

Linear regression analyses were performed on the numerical variables to analyse the effect of each factor on compliance with the CPG recommendations (outcome measures for these analyses were “Overall adherence to PU recommendations” and “Overall adherence to vascular access care recommendations”, see Additional file 2). A value of *p* < 0.05 was assumed to indicate statistical significance. IBM-SPSS v.26 statistical software was used for all these analyses.

### Validity and reliability/rigour

The study variables were measured by direct observation, by nurses specifically trained to ensure the homogeneity of the data obtained. The questionnaires used in selecting the units (Practice Environment Scale-Nursing Work Index) and nurses (Advanced Practice Nursing Competency Assessment Instrument) included in the study were validated in Spanish and adapted to the context. The Cronbach’s α values of these instruments, in the validated version, were 0.91 and 0.96, respectively.

## Results

### Participants

In total, 6373 audits were carried out. In the case of pressure ulcer audits, 3742 patients were included, of whom 1797 were in the intervention group and 1945 in the control group. By sex, 2094 patients were male and 1648 were female. The mean (SD) age of the sample was 68.2 years ±16.6. Regarding the patients included in the audit of vascular access devices, the study sample was 2631 patients, 1290 in the intervention group and 1341 in the control group, of whom 1493 were male and 1138 were female. The mean (SD) age of the sample was 70.7 ± 16.3 years. No differences were observed in the distribution by sex between the study groups (Table 1).

**Table 1:** Demographic characteristics of participants

**Patients included in pressure ulcers assessment**		
	Intervention group	Control group
Admision unit, n (%)	1797 (48,02%)	1945 (51,98%)
Male Gender, n (%)	984 (54.76%)	1110 (57.07%)
Age, mean (SD) (years)	67.3 ± 16.2	69 ± 16.9
**Patients included in vascular access devices assessment**		
	Intervention	Control
Admision unit, n (%)	1290 (49.03%)	1341 (50.96%)
Male Gender, n (%)	736 (57.05%)	757 (56.45%)
Age, mean (SD) (years)	69.2 ± 16.5	72.2 ± 15.9

### Indicators derived from the clinical practice guidelines for pressure ulcers

An additional file shows the monthly evolution of the variables observed for these CPG, for both groups (see Additional file 3).

**Fig. 1:**
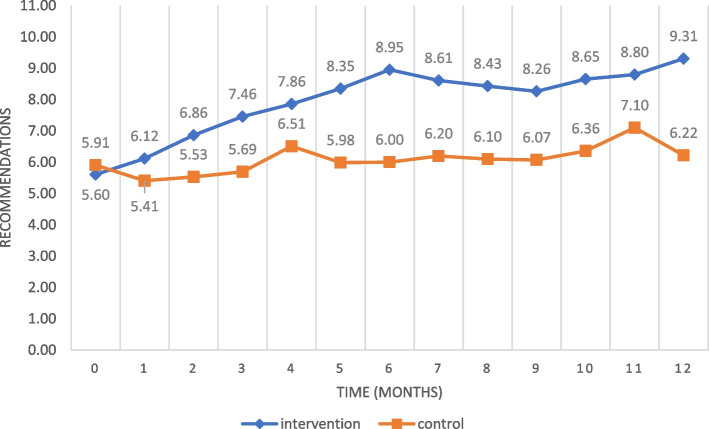
Mean adherence to the CPG recommendations for the prevention and treatment of pressure ulcers

The number of patients at risk or with pressure ulcers present, and therefore candidates to receive care based on CPG recommendations, ranged between 30 and 40% of all patients audited each month (a total of 1329 patients, 567 in the intervention group and 762 in the control group). Table 2 shows the percentage of compliance with these recommendations in each unit at the beginning and the end of the intervention period. Figure 1 shows the evolution of adherence to the eleven process indicators measured monthly.

**Table 2:** Compliance with the CPG recommendations for the prevention and treatment of pressure ulcers

	Baseline (pre-intervention)			12 months (post-intervention)			Within-group pre-post difference	
	APHN	Control	*p* value	APHN	Control	*p* value	APHN (*p* value)	Control (*p* value)
PU risk assessment among candidates for extended care	65%	60%	0.633	94%	62%	<.001	29 pp. (0.000)	1 pp. (0.878)
PU risk reassessment	12%	13%	0.816	83%	16%	<.001	72 pp. (0.000)	3 pp. (0.644)
Daily assessment of skin condition	7%	2%	0.322	69%	22%	<.001	62 pp. (0.000)	20 pp. (0.001)
Barrier / moisturiser cream or oil	72%	70%	0.807	89%	47%	<.001	17 pp. (0.064)	−23 pp. (0.018)
Daily record of skin condition	0%	43%	0.000	97%	36%	<.001	97 pp. (0.000)	−7 pp. (0.455)
Postural changes scheduled	67%	55%	0.205	92%	67%	0.007	24 pp. (0.009)	13 pp. (0.181)
Pressure modification/Pressure relief support (PMS/PRS)	93%	87%	0.504	92%	89%	1.000	−1 pp. (1.000)	2 pp. (0.714)
Nutritional assessment	21%	30%	0.304	33%	16%	0.060	12 pp. (0.214)	−14 pp. (0.089)
Full record of PU characteristics	84%	83%	0.927	100%	95%	0.275	16 pp. (0.014)	12 pp. (0.057)
PU treatment schedule	81%	87%	0.469	94%	93%	1.000	13 pp. (0.101)	6 pp. (0.308)
Patients’ and families’ understanding of condition	58%	60%	0.824	86%	78%	0.343	28 pp. (0.006)	18 pp. (0.045)

*PU:* Pressure ulcer; *APHN* Advanced practice hospitalization nurse; *pp*. Percentage point

Table 3 shows the results of the linear regression model created to determine the influence of the intervention on adherence to the CPG recommendations for the prevention and care of pressure ulcers among patients who were candidates for extended care (*n* = 1329). As observed, adherence to the CPG recommendations arise more likely to happen if the patient is admitted to an APHN unit as intervention time progresses. The patient’s risk of pressure ulcer does not affect adherence to recommendations.

**Table 3:** Variables that influence overall adherence to pressure ulcers recommendations

*N* = 1329	Simple linear regression				Multiple linear regression			
	B	95% CI		***p*** value	B	95% CI		***p*** value
**Unit (APHN / control)**	1.833	1.635	2.031	0.000	1.865	1.677	2.053	<.001
**Time (months)**	0.151	0.123	0.179	0.000	0.156	0.131	0.181	<.001
**Risk of PU**^**†**^	−0.072	− 0.307	0.164	0.551	0.094	−0.106	0.295	0.357

*PU:* pressure ulcer; *APHN* Advanced practice hospitalisation nurse; *CI* Confidence interval

^**†**^ Patient at risk of developing a pressure ulcer was defined as one presenting a Braden score < 17 points in patients younger than 75 years or < 19 points in patients older than 75 years

### Indicators derived from the clinical practice guidelines for vascular access devices

An additional file shows the monthly evolution of the variables observed in each of the study groups (see Additional file 4).

**Fig. 2:**
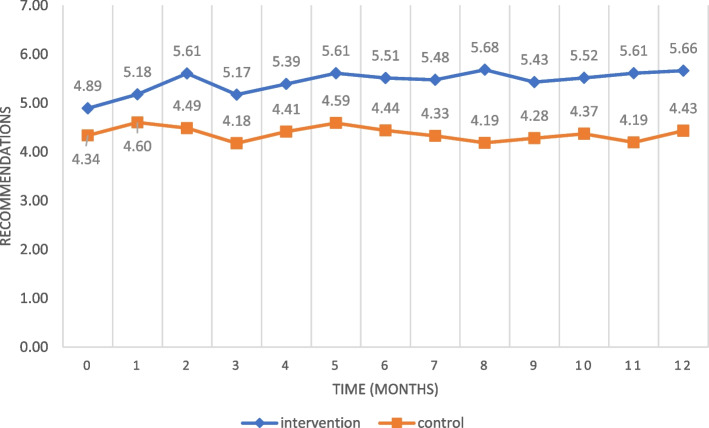
Mean adherence to the CPG recommendations for the insertion and maintenance of vascular access devices

Table 4 shows the percentage of compliance with the CPG recommendations for vascular access in each unit, before and after the intervention. The evolution of adherence to the seven process indicators, measured monthly, is shown in Fig. 2.

**Table 4:** Compliance with the recommendations for the care of peripheral catheters

	Baseline (pre-intervention)			12 months (post-intervention)			Within-group pre-post difference	
	APHN	Control	p value	APHN	Control	p value	APHN (p value)	Control (p value)
Catheters inserted in the correct location	84%	78%	0.279	78%	73%	0.363	−6pp (0.266)	−6pp (0.372)
Catheters inserted, with the orifice visible	67%	58%	0.193	90%	71%	<.001	23pp (0.000)	13pp (0.066)
Catheters in use	85%	92%	0.120	89%	83%	0.227	4pp (0.416)	−9pp (0.055)
Duration of insertion	66%	53%	0.078	60%	47%	0.067	−5pp (0.435)	−6pp(0.420)
Type of attachment	67%	57%	0.146	88%	69%	<.001	21pp (0.000)	13pp (0.066)
Condition of the dressing	60%	59%	0.875	75%	67%	0.222	15pp (0.019)	9pp (0.219)
Catheter record	61%	37%	0.001	85%	33%	<.001	24pp (0.000)	−4pp (0.535)

*APHN:* Advanced practice hospitalisation nurse, *pp.* Percentage point

Table 5 shows the results of the linear regression model created to determine the influence of the intervention on adherence to CPG recommendations for the insertion and care of vascular access devices. Again, the CPG recommendations are more likely to be followed if the patient is admitted to an APHN unit as intervention time progresses.

**Table 5:** Variables that influence overall adherence to vascular access recommendations

*N* = 2631	Simple linear regression				Multiple linear regression			
	B	95% CI		*p* value	B	95% CI		*p* value
**Unit (APHN / Control)**	1.068	0.955	1.180	0.000	1.066	0.953	1.178	<.001
**Time (months)**	0.016	0.000	0.032	0,048	0.014	−0.001	0.029	0.077

*APHN:* Advanced practice hospitalisation nurse; *CI* Confidence interval

## Discussion

To our knowledge, the present study is the first that has been conducted to measure the impact produced on indicators of clinical results by the presence of an APHN in the hospital unit when these indicators depend directly on the nursing care provided. The study results revealed a significant improvement in most of the clinical indicators derived from both the CPGs of pressure ulcer prevention and treatment and vascular access (catheterisation) care and maintenance (Tables 2 and 4), especially those related to the nursing care process. Higher adherence to evidence-based recommendations is observed in those units led by APHNs.

The findings we report are in line with those obtained in previous studies regarding the positive influence of APNs on clinical outcomes [46]. Many of these earlier studies compared the results of patients seen by APNs with those supervised by other healthcare providers, concluding that the outcomes for the former were equally good or even better [46–48]. The incorporation of APNs into multidisciplinary teams not only improves health system results but also reduces costs [49]. However, it is sometimes difficult to measure the specific effect of an APN’s contribution, precisely because this professional forms part of a multidisciplinary team [50]. Our study addresses this very question, by measuring clinical indicators that depend directly on the nursing input, thus reflecting the individual’s contribution and impact on patient care.

Analysis of the process indicators and of the linear regression suggests that the improvement in adherence to CPGs recommendations is produced by the direct intervention of the APHNs. The magnitude of this improvement increased as the intervention time progressed, and the curve never completely flattened during the intervention. Therefore, the long-term impact of the intervention remains to be determined.

In the specific case of the pressure ulcer guideline, adherence to the CPG recommendations and the improvements observed were independent of the patient’s level of risk. This observation corroborates previous studies according to which awareness of CPGs is not sufficient to ensure compliance. Instead, a broad range of factors that may produce behavioural change must be taken into account [29, 51, 52].

Prior research has shown that the contribution made by APNs improves pressure ulcer care and decreases its prevalence [53]. In our case, the pre-post analysis of the process and outcome indicators of pressure ulcers showed that almost all aspects of nursing care related to the treatment and prevention of these injuries in patients at risk were significantly improved in the intervention group, compared to the control group. Among the patients in the intervention group, almost 91% received a risk assessment on admission to hospital (an increase of 22 percentage points over the previous situation), which contrasts with the 62% measured in the control group (an 11 percentage points improvement). In the intervention group, moreover, we also observed a 30 percentage points increase in risk reassessment when this was indicated, an effect that was not observed in the control group. The adherence to the different variables at the beginning of the study was heterogeneous, some of them showing initial high compliance (ie. use of pressure modification/pressure relief support surfaces, record of PU characteristics or treatment schedule) possibly due to environmental or organisational characteristics that influence clinical staff to be more sensitive to certain aspects of care [54].

With respect to the impact of APHN intervention on compliance with the CPG recommendations for the use and maintenance of vascular access devices, we observed a reduction in catheter-related adverse events in both study groups, but they were statistically significantly less prevalent in the intervention group (9%) than in the control group (20%). This question is of great importance to patients’ health, as reducing adverse events is directly associated with preventing bacteremia. Moreover, it considerably cuts health care costs [55, 56].

In our study, statistically significant improvements were obtained in the care and maintenance of catheters, in the variables concerning visual inspection of the insertion orifice, in the type of catheter attachment employed and in the records kept of catheter characteristics. The duration of catheter use tended to be greater in the intervention group. This might be explained by the closer monitoring performed of the device status, which helped achieve lower rates of adverse events, despite the prolonged insertion of the catheters [57].

There were no statistically significant improvements in all the other indicators considered. The variable “location of the catheter” was less influenced by the intervention, probably due to the complexity of measuring the long-term suitability of a given location. In addition, many patients are admitted with the catheter already in place, it having been inserted during previous attention, in emergency department or any different unit. This situation may influence the assessment of the catheter suitability, as it may vary from when the catheter was originally inserted until the time at which the audit is performed.

Another notable result obtained is the increased number of nursing records kept related to the two processes under study. Although these figures increased overall, the increase was sharper in the intervention unit. Clinical documentation is an essential part of nursing care [58], by facilitating access to valid patient data and enabling healthcare providers to make a timely evaluation and deliver appropriate follow-up [59].

Although the contribution made by APNs has been studied in various settings, ranging from primary to specialised care, using indicators that directly reflect APN care [46, 48, 49, 60] the role and importance of these professionals in the Spanish health system have yet to be fully determined. Thus, while some institutions have incorporated nurses with advanced skills, especially in the care of patients with chronic diseases [50, 60], their status is neither well known nor formally regulated [61]. Our study shows that the incorporation of APHNs enhances clinical outcomes, and therefore it would be useful to conduct cost-effectiveness studies in this field, as has been proposed elsewhere [47, 49, 50, 62]. In our view, the improved quality of care and the cost reductions facilitated by the introduction of APHNs into hospital units are undeniable and should be acknowledged by policymakers when the opportunity arises to incorporate these new roles into the health system [62].

Working to improve healthcare practices is a complex, multifaceted task, and therefore any improvement strategy proposed should be carefully piloted before implementation [1]. The present study represents an initial step towards understanding this process within a still incipient context in the field of advanced healthcare practice [41]. APHNs must function within the framework of models of proven efficiency. In line with previous research, our study highlights the role of APNs as agents of change, stimulating evidence-based practices, and facilitating the implementation of the CPG recommendations in hospitals’ nursing teams [9, 24–26, 63].

### Limitations

This study is subject to certain limitations, especially the fact that the indicators were not monitored continuously, but were evaluated on predetermined, non-randomised days. Moreover, while the study variables were evaluated by direct observation of the care performed by the unit nurses, slight improvements were also observed in the control units, a phenomenon which could be explained by the Hawthorne effect, since it is difficult to maintain usual work behaviour when the teams are aware that they are being studied [64]. Furthermore, as similar units from the same hospital were selected for study, there is the possibility that some of the dynamics established in an intervention unit may have influenced the actions of those working in the control unit. Finally, there may have been some interchange of nurses between the intervention and control units, which would also have affected the behaviour patterns observed.

## Conclusions

The incorporation of Advanced Practice Hospitalisation Nurses (APHNs) into hospital units significantly improves clinical indicators related to the prevention and treatment of pressure ulcers and the insertion and maintenance of peripheral catheters. Overall, these effects are reflected as a decrease in the number of adverse events experienced. Our study describes the specific contributions made by APHNs in terms of the health indicators considered. Our focus on two CPGs illustrates the potential benefits of incorporating APHNs into hospital units, in terms of the implementation of evidence and fostering adherence to the CPGs among other members of the nursing team. The results we present highlight the need to consider modifying certain professional roles, adapting them to new models of proven efficacy.

## Supplementary Information

**Additional file 1.**

**Additional file 2.**

**Additional file 3.**

**Additional file 4.**

## Data Availability

The datasets generated and/or analysed during the current study are not publicly available due to privacy and confidentiality reasons, but are available from the corresponding author on reasonable request.

## Abbreviations

CPG: Clinical Practice Guideline
APN: Advanced Practice Nurse
APHN: Advanced Practice Hospitalization Nurse.
