# Supplementary material for: Impact of advanced practice nurses in hospital units on compliance with clinical practice guidelines: a quasi-experimental study
Source: BMC Nurs. 2022 Nov 29;21:331. doi: 10.1186/s12912-022-01110-x (PMC9706842; doi:10.1186/s12912-022-01110-x)
Supplement: Supplementary file 1 — Additional file 1. [file 12912_2022_1110_MOESM1_ESM.pdf]

Additional file 1. Nursing Work Index values obtained prior to the intervention

	Intervention units (n=60)	Control units (n=52)	p value
Nurse Participation	55.8%	51.0%	0.26
Nursing Foundations	57.0%	60.2%	0.27
Leadership Ability	84.2%	83.8%	0.89
Staffing and Resource Adequacy	51.3%	42.7%	0.02
Nurse-Physician Relationships	53.4%	50.6%	0.43

n: Number of nurses evaluated in each unit before the intervention
