# Supplementary material for: Impact of advanced practice nurses in hospital units on compliance with clinical practice guidelines: a quasi-experimental study
Source: BMC Nurs. 2022 Nov 29;21:331. doi: 10.1186/s12912-022-01110-x (PMC9706842; doi:10.1186/s12912-022-01110-x)
Supplement: Supplementary file 2 — Additional file 2. [file 12912_2022_1110_MOESM2_ESM.pdf]

Additional File 2. Specific indicators within the CPGs

Variable	Description	Definition
**Pressure ulcer process indicators**		
**Risk of PU**	Patients at risk of PU in the target population, according to the Braden scale*	Number of patients at risk of PU according to the Braden scale x 100 /Number of hospitalised patients
**Candidates for extended care**	Patients at risk of PU according to the Braden scale*, or with present PU	Number of patients at risk of PU according to the Braden scale or with present PU x 100 /Number of hospitalised patients
**PU risk assessment**	Application of the Braden scale on admission to the unit, to assess the risk of PU	Number of patients at risk according to the Braden scale x 100 / Number of hospitalised patients
**PU risk assessment among candidates for extended care**	Application of the Braden scale on admission to the unit, to assess the risk of PU in patients at risk of PU[†], or with present PU.	Number of patients at risk according to the Braden scale x 100 /Number of hospitalised patients at risk of PU or with present PU.
**PU risk reassessment**	Braden scale assessment of patients at risk according to risk level[†]: every 24 hours if high risk, every 72 hours if moderate risk, weekly if low risk. In all cases of PU risk or presence, reassess if relevant clinical change occurs.	Number of patients at risk according to the Braden scale, reassessed according to level of risk or need x 100 / Number of hospitalised patients at risk of PU or with present PU.
**Daily assessment of the skin**	Daily assessment of the skin among candidates for extended care	Number of patients whose skin condition is assessed daily x100 / Number of hospitalised patients at risk of PU or with present PU.
**Barrier / moisturiser cream or oil**	Use of moisturising creams or oils in PU risk areas among candidates for extended care.	Number of patients given oils or moisturisers in areas at risk of PU x100 / Number of hospitalised patients at risk of PU or with present PU.
**Daily record of skin condition**	A record of skin condition is maintained for patients who are candidates for extended care.	Number of patients included in the skin condition register x100 / Number of hospitalised patients at risk of PU or with present PU.
**Postural changes scheduled**	An appropriate schedule of postural changes is maintained for patients who are candidates for extended care and who require total or partial help. No such schedule is maintained for patients who do not require this assistance.	Number of patients with an appropriate schedule of postural changes x100 / Number of hospitalised patients at risk of PU or with present PU.

**Pressure modification/Pressure relief support (PM/PRS)**	Use of an appropriate pressure relief mechanism or surface, depending on the level of risk: active surface for patients at high risk of PU[†] or who present stage 3 or 4 PU, passive surface otherwise.	Number of patients using pressure relief mechanism or surface according to the risk level of PU x100 / Number of hospitalised patients at risk of PU or with present PU.
**Nutritional assessment**	A nutritional assessment is performed for patients who are candidates for extended care.	Number of patients who receive nutritional assessment x100 / Number of hospitalised patients at risk of PU or with present PU.
**Full record of PU characteristics**	If the patient presents a PU when the audit is performed, a record is kept of the characteristics of the PU, including origin, location and category. If there is no PU, no record is kept.	Number of patients for whom a record of PU characteristics is kept x100 / Number of hospitalised patients at risk of PU or with present PU.
**PU treatment schedule**	If the patient presents a PU when the audit is performed, a record is kept of the specific care regimen prescribed for PU and its frequency of application. If there is no PU, no record is kept.	Number of patients for whom a record of the PU care regimen is kept x100 / Number of hospitalised patients at risk of PU or with present PU.
**Patients' and families' understanding of condition**	Patients who are candidates for extended care are aware of what a PU is and how to prevent it (unless there is cognitive impairment or a language barrier).	Number of patients informed about PUs x100 / Number of hospitalised patients at risk of PU or with present PU.
**Overall adherence to PU recommendations**	Average percent score for the 11 PU process variables: PU risk assessment, PU risk assessment among candidates for extended care, PU risk reassessment, Daily assessment of the skin, Use of barrier/moisturiser cream or oil, Daily record of skin condition, Postural changes scheduled, PM/PRS, Nutritional assessment, Full record of PU characteristics, PU treatment schedule, Patients' and families' understanding of condition.	Mean number of recommendations fulfilled for each patient x100 / Total number of PU guideline recommendations.
**Pressure ulcer outcome indicators**		
**Prevalence of PU**	Proportion of patients hospitalised with PU	Number of patients with PU x100/ Number of hospitalised patients
**Prevalence of PU among patients at risk**	Proportion of patients at risk[†] who present PU	Number of patients with PU x100/ Number of patients at risk during the study period
**Incidence of PU**	Number of new cases of PU	Number of patients with PU x100/ Cumulative number of hospitalised patients

**Incidence of PU among patients at risk**	Number of new cases of PU among patients at risk[†]	Number of patients with PU x100/ Cumulative number of patients at risk during the study period
**Other skin lesions**	Presence of skin lesions other than PU among patients who are candidates for extended care	Number of patients with other skin lesions x100/ Number of patients at risk during the study period
**Vascular access devices process indicators**		
**Catheters inserted in the antecubital fossa**	If a catheter must be inserted in the upper limbs, it should be in the antecubital fossa.	Number of catheters inserted in the antecubital fossa x100/ Number of catheters inserted in upper limbs
**Catheters inserted in the lower limbs**	The catheter is inserted in the lower limb.	Number of catheters inserted in lower limbs x100/ Total number of catheters
**Record of catheters inserted in the antecubital fossa**	If there is a record of catheter insertion in the upper limbs, insertion in the antecubital fossa is specified.	Number of catheters recorded as inserted in the antecubital fossa x100/ Number of catheters recorded as inserted in upper limbs
**Record of catheters inserted in the lower limbs**	Insertion of the catheter in the lower limb is recorded.	Number of catheters recorded as inserted in lower limbs x100/ Total number of catheters recorded as inserted
**Record of catheters inserted, with the orifice visible**	If the catheter insertion is recorded, the orifice is visible.	Number of catheters recorded as inserted, with the orifice visible to the naked eye x100/ Total number of catheters recorded as inserted
**Catheters inserted in a recommended location**	The catheter is inserted in a recommended location, avoiding the antecubital fossa, inner wrist and lower limbs.	Number of catheters inserted in a recommended location (avoiding the antecubital fossa, inner wrist and lower limbs) x100/ Total number of catheters inserted
**Catheters inserted, with the orifice visible**	The orifice of the catheter insertion is visible.	Number of catheters inserted, with the orifice visible to the naked eye x100/ Total number of catheters
**Catheters in use**	The catheter has been used in the last 24 hours.	Number of catheters used in the last 24 hours x100/ Total number of catheters inserted
**Duration of catheter insertion**	The catheter has been inserted for less than 96 hours.	Number of catheters inserted less than 96 hours ago x100/ Total number of catheters inserted
**Type of catheter attachment**	The catheter attachment used facilitates assessment and monitoring of the insertion site, and inhibits the extravasation or displacement of the catheter.	Number of catheters fitted with an attachment that facilitates assessment and monitoring of the insertion site, and inhibits the extravasation or displacement of the catheter x100/ Total number of catheters inserted
**Condition of the dressing**	The dressing is clean, dry and intact, leaving the catheter insertion orifice visible.	Number of catheters with a clean, dry, intact dressing x100/ Total number of catheters inserted

**Catheter record**	The use of the catheter is recorded.	Number of catheters recorded as inserted x100/ Total number of catheters inserted
**Overall adherence to vascular access care recommendations**	Average percent score for the 7 vascular access process variables: Correct location, Catheters in use, Duration of catheter insertion, Type of attachment, Condition of the dressing, Catheter record.	Mean number of recommendations fulfilled for each patient x100 / Total number of VA recommendations.
**Vascular access devices outcome indicators**		
**Prevalence of adverse events**	Proportion of patients experiencing peripheral catheter-related adverse events during on-site assessment.	Number of adverse events x100/ Number of hospitalised patients
**Incidence of adverse events**	Incidence of patients experiencing peripheral catheter-related adverse events during on-site evaluation	Number of adverse events x100 / Number of hospitalised patients
**Erythema**	Percentage of catheters inserted with visible insertion site and presenting erythema at the orifice.	Number of patients with erythema x100 / Number of catheters inserted with a visible insertion orifice.
**Venous cord**	The patient has a venous cord in the catheter tract, in catheters with a visible orifice.	Number of patients with a venous cord x100 / Number of catheters inserted with a visible insertion orifice.
**Inflammation**	The patient has inflammation in the catheter area, in catheters with a visible orifice.	Number of patients with inflammation x100 / Number of catheters inserted with a visible insertion orifice.
**Pain**	The patient reports a score of 1 or more on the VAS pain scale.	Number of patients with pain x100 /Number of hospitalised patients.
**Unclassified adverse event**	The patient presents an adverse event that cannot be classified among the above, in catheters with a visible orifice.	Number of patients with an unclassified adverse event x100 / Number of patients presenting an adverse event for assessment.
**Unnecessary catheters**	Catheters not used in the last 24 hours.	Number of patients with a catheter inserted during the last 24 hours but not used x100 / Number of hospitalised patients.

PU: Pressure ulcer; PM/PRS: Pressure modification / pressure relief support.

† Risk levels according to the Braden scale: High risk 12 points or less; Moderate risk 13-14 points; Low risk 15-16 points in patients aged <75 years or 15-18 points in patients > 75 years; No risk 17-23 points in patients aged <75 years or 19-23 points in patients aged >75 years.
