# Supplementary material for: Impact of advanced practice nurses in hospital units on compliance with clinical practice guidelines: a quasi-experimental study
Source: BMC Nurs. 2022 Nov 29;21:331. doi: 10.1186/s12912-022-01110-x (PMC9706842; doi:10.1186/s12912-022-01110-x)
Supplement: Supplementary file 3 — Additional file 3. [file 12912_2022_1110_MOESM3_ESM.pdf]

Additional File 3. Evolution of the process and outcome indicators derived from the CPG for pressure ulcers

Pressure ulcer process indicators															
		**Baseline**	**Month 1**	**Month 2**	**Month 3**	**Month 4**	**Month 5**	**Month 6**	**Month 7**	**Month 8**	**Month 9**	**Month 10**	**Month 11**	**Month 12**	**Overall**
**Risk of PU**	**APHN**	41/151 (27.2%)	47/125 (37.6%)	35/130 (26.9%)	44/134 (32.8%)	42/139 (30.2%)	37/141 (26.2%)	42/131 (32.1%)	46/138 (33.3%)	42/141 (29.8%)	38/145 (26.2%)	47/149 (31.5%)	46/147 (31.3%)	38/126 (30.2%)	545/1797 (30.3%)
	**Control**	53/160 (33.1%)	55/153 (35.9%)	53/153 (34.6%)	61/156 (39.1%)	54/146 (37.0%)	57/146 (39.0%)	58/141 (41.1%)	52/144 (36.1%)	50/139 (36.0%)	69/146 (47.3%)	66/153 (43.1%)	60/157 (38.2%)	55/151 (36.4%)	743/1945 (38.2%)
		p=0.236	p=0.776	p=0.162	p=0.268	p=0.226	p=0.020	p=0.121	p=0.624	p=0.270	p=0.000	p=0.037	p=0.205	p=0.271	**p=0.000**
**Candidates for extended care**	**APHN**	43/151 (28.5%)	52/125 (41.6%)	36/130 (27.7%)	46/134 (34.3%)	42/139 (30.2%)	43/141 (30.5%)	41/131 (31.3%)	46/138 (33.3%)	42/141 (29.8%)	42/145 (29.0%)	49/149 (32.9%)	49/147 (33.3%)	36/126 (28.6%)	567/1797 (31.6%)
	**Control**	53/160 (33.1%)	54/153 (35.3%)	55/153 (35.9%)	62/156 (39.7%)	55/146 (37.7%)	63/146 (43.2%)	61/141 (43.3%)	56/144 (38.9%)	51/139 (36.7%)	70/146 (47.9%)	67/153 (43.8%)	60/157 (38.2%)	55/151 (36.4%)	762/1945 (39.2%)
		p=0.375	p=0.282	p=0.138	p=0.342	p=0.184	p=0.026	p=0.042	p=0.332	p=0.220	p=0.001	p=0.051	p=0.375	p=0.166	**p=0.000**
**PU risk assessment**	**APN**	88/151 (58.3%)	66/125 (52.8%)	99/130 (76.2%)	119/134 (88.8%)	120/139 (86.3%)	122/141 (86.5%)	102/131 (77.9%)	116/138 (84.1%)	124/141 (87.9%)	126/145 (86.9%)	125/149 (83.9%)	127/147 (86.4%)	114/126 (90.5%)	1448/1797 (80.6%)
	**Control**	81/160 (50.6%)	70/153 (45.8%)	90/153 (58.8%)	93/156 (59.6%)	83/146 (56.8%)	76/146 (52.1%)	86/141 (61.0%)	69/144 (47.9%)	84/139 (60.4%)	89/146 (61.0%)	94/153 (61.4%)	104/157 (66.2%)	93/151 (61.6%)	1113/1945 (57.2%)
		p=0.176	p=0.242	p=0.002	p=0.000	p=0.000	p=0.000	p=0.004	p=0.000	p=0.000	p=0.000	p=0.000	p=0.000	p=0.000	**p=0.000**
**PU risk assessment among candidates for extended care**	**APHN**	28/43 (65.1%)	28/52 (53.8%)	29/36 (80.6%)	44/46 (95.7%)	39/42 (92.9%)	40/43 (93.0%)	37/41 (90.2%)	38/46 (82.6%)	34/42 (81.0%)	35/42 (83.3%)	41/49 (83.7%)	42/49 (85.7%)	34/36 (94.4%)	469/567 (82.7%)
	**Control**	32/53 (60.4%)	26/54 (48.1%)	38/55 (69.1%)	43/62 (69.4%)	35/55 (63.6%)	35/63 (55.6%)	40/61 (65.6%)	27/56 (48.2%)	30/51 (58.8%)	51/70 (72.9%)	49/67 (73.1%)	46/60 (76.7%)	34/55 (61.8%)	486/762 (63.8%)
		p=0.633	p=0.557	p=0.225	p=0.001	p=0.001	p=0.000	p=0.005	p=0.000	p=0.022	p=0.204	p=0.179	p=0.233	p=0.000	**p=0.000**
**PU risk reassessment**	**APHN**	5/43 (11.6%)	17/52 (32.7%)	19/36 (52.8%)	26/46 (56.5%)	28/42 (66.7%)	30/43 (69.8%)	35/41 (85.4%)	32/46 (69.6%)	28/42 (66.7%)	35/42 (83.3%)	40/49 (81.6%)	39/49 (79.6%)	30/36 (83.3%)	364/567 (64.2%)

	**Control**	7/53 (13.2%)	2/54 (3.7%)	3/55 (5.5%)	4/62 (6.5%)	6/55 (10.9%)	4/63 (6.3%)	1/61 (1.6%)	3/56 (5.4%)	5/51 (9.8%)	3/70 (4.3%)	7/67 (10.4%)	8/60 (13.3%)	9/55 (16.4%)	62/762 (8.1%)
		p=0.816	p=0.000	p=0.000	p=0.000	p=0.000	p=0.000	p=0.000	p=0.000	p=0.000	p=0.000	p=0.000	p=0.000	p=0.000	**p=0.000**
**Daily assessment of the skin**	**APHN**	3/43 (7.0%)	3/52 (5.8%)	6/36 (16.7%)	4/46 (8.7%)	6/42 (14.3%)	21/43 (48.8%)	30/41 (73.2%)	26/46 (56.5%)	19/42 (45.2%)	16/42 (38.1%)	31/49 (63.3%)	23/49 (46.9%)	25/36 (69.4%)	213/567 (37.6%)
	**Control**	1/53 (1.9%)	1/54 (1.9%)	2/55 (3.6%)	1/62 (1.6%)	6/55 (10.9%)	13/63 (20.6%)	16/61 (26.2%)	14/56 (25.0%)	12/51 (23.5%)	15/70 (21.4%)	18/67 (26.9%)	16/60 (26.7%)	12/55 (21.8%)	127/762 (16.7%)
		p=0.322	p=0.358	p=0.054	p=0.161	p=0.617	p=0.002	p=0.000	p=0.001	p=0.027	p=0.056	p=0.000	p=0.028	p=0.000	**p=0.000**
**Barrier / moisturiser cream or oil**	**APHN**	31/43 (72.1%)	30/52 (57.7%)	26/36 (72.2%)	25/46 (54.3%)	36/42 (85.7%)	25/43 (58.1%)	33/41 (80.5%)	37/46 (80.4%)	33/42 (78.6%)	29/42 (69.0%)	38/49 (77.6%)	43/49 (87.8%)	32/36 (88.9%)	418/567 (73.7%)
	**Control**	37/53 (69.8%)	21/54 (38.9%)	27/55 (49.1%)	30/62 (48.4%)	36/55 (65.5%)	31/63 (49.2%)	27/61 (44.3%)	37/56 (66.1%)	34/51 (66.7%)	39/70 (55.7%)	47/67 (70.1%)	47/60 (78.3%)	26/55 (47.3%)	439/762 (57.6%)
		p=0.807	p=0.053	p=0.029	p=0.540	p=0.024	p=0.366	p=0.000	p=0.106	p=0.203	p=0.162	p=0.374	p=0.197	p=0.005	**p=0.000**
**Daily record of skin condition**	**APHN**	0/43 (0.0%)	6/52 (11.5%)	8/36 (22.2%)	25/46 (54.3%)	19/42 (45.2%)	28/43 (65.1%)	32/41 (78.0%)	32/46 (69.6%)	26/42 (61.9%)	25/42 (59.5%)	38/49 (77.6%)	41/49 (83.7%)	35/36 (97.2%)	315/567 (55.6%)
	**Control**	23/53 (43.4%)	23/54 (42.6%)	17/55 (30.9%)	17/62 (27.4%)	27/55 (49.1%)	25/63 (39.7%)	21/61 (34.4%)	16/56 (28.6%)	26/51 (51.0%)	28/70 (40.0%)	25/67 (37.3%)	32/60 (53.3%)	20/55 (36.4%)	300/762 (39.4%)
		p=0.000	p=0.000	p=0.364	p=0.005	p=0.707	p=0.010	p=0.000	p=0.000	p=0.291	p=0.045	p=0.000	p=0.001	p=0.000	**p=0.000**
**Postural changes scheduled**	**APHN**	29/43 (67.4%)	40/52 (76.9%)	27/36 (75.0%)	39/46 (84.8%)	39/42 (92.9%)	38/43 (88.4%)	39/41 (95.1%)	41/46 (89.1%)	34/42 (81.0%)	37/42 (88.1%)	42/49 (85.7%)	41/49 (83.7%)	33/36 (91.7%)	479/567 (84.5%)
	**Control**	29/53 (54.7%)	30/54 (55.6%)	28/55 (50.9%)	34/62 (54.8%)	40/55 (72.7%)	38/63 (60.3%)	40/61 (65.6%)	45/56 (80.4%)	27/51 (52.9%)	39/70 (55.7%)	43/67 (64.2%)	44/60 (73.3%)	37/55 (67.3%)	474/762 (62.2%)
		p=0.205	p=0.020	p=0.022	p=0.001	p=0.012	p=0.002	p=0.000	p=0.225	p=0.005	p=0.000	p=0.010	p=0.195	p=0.007	**p=0.000**
**Pressure modification/Press**	**APHN**	40/43 (93.0%)	47/52 (90.4%)	33/36 (91.7%)	45/46 (97.8%)	38/42 (90.5%)	43/43 (100.0%)	39/41 (95.1%)	42/46 (91.3%)	41/42 (97.6%)	41/42 (97.6%)	47/49 (95.9%)	48/49 (98.0%)	33/36 (91.7%)	537/567 (94.7%)

**ure relief support (PM/PRS)**	**Control**	46/53 (86.8%)	51/54 (94.4%)	48/55 (87.3%)	47/62 (75.8%)	47/55 (85.5%)	51/63 (81.0%)	51/61 (83.6%)	46/56 (82.1%)	45/51 (88.2%)	59/70 (84.3%)	53/67 (79.1%)	54/60 (90.0%)	49/55 (89.1%)	647/762 (84.9%)
		p=0.320	p=0.484	p=0.512	p=0.001	p=0.457	p=0.002	p=0.077	p=0.181	p=0.123	p=0.027	p=0.009	p=0.126	p=0.687	**p=0.000**
**Nutritional assessment**	**APHN**	9/43 (20.9%)	7/52 (13.5%)	4/36 (11.1%)	10/46 (21.7%)	8/42 (19.0%)	15/43 (34.9%)	7/41 (17.1%)	17/46 (37.0%)	19/42 (45.2%)	13/42 (31.0%)	8/49 (16.3%)	13/49 (26.5%)	12/36 (33.3%)	142/567 (25.0%)
	**Control**	16/53 (30.2%)	3/54 (5.6%)	3/55 (5.5%)	9/62 (14.5%)	8/55 (14.5%)	9/63 (14.3%)	3/61 (4.9%)	3/56 (5.4%)	6/51 (11.8%)	9/70 (12.9%)	8/67 (11.9%)	9/60 (15.0%)	9/55 (16.4%)	95/762 (12.5%)
		p=0.304	p=0.197	p=0.428	p=0.330	p=0.554	p=0.013	p=0.085	p=0.000	p=0.000	p=0.020	p=0.499	p=0.136	p=0.060	**p=0.000**
**Full record of PU characteristics**	**APHN**	36/43 (83.7%)	50/52 (96.2%)	35/36 (97.2%)	46/46 (100.0%)	42/42 (100.0%)	42/43 (97.7%)	40/41 (97.6%)	46/46 (100.0%)	42/42 (100.0%)	41/42 (97.6%)	47/49 (95.9%)	48/49 (98.0%)	36/36 (100.0%)	551/567 (97.2%)
	**Control**	44/53 (83.0%)	47/54 (87.0%)	52/55 (94.5%)	59/62 (95.2%)	55/55 (100.0%)	60/63 (95.2%)	59/61 (96.7%)	53/56 (94.6%)	46/51 (90.2%)	64/70 (91.4%)	64/67 (95.5%)	58/60 (96.7%)	52/55 (94.5%)	713/762 (93.6%)
		p=1.000	p=0.161	p=1.000	p=0.260	p=1.000	p=0.645	p=1.000	p=0.250	p=0.062	p=0.253	p=1.000	p=1.000	p=0.275	**p=0.003**
**PU treatment schedule**	**APHN**	35/43 (81.4%)	50/52 (96.2%)	35/36 (97.2%)	46/46 (100.0%)	42/42 (100.0%)	42/43 (97.7%)	40/41 (97.6%)	46/46 (100.0%)	42/42 (100.0%)	37/42 (88.1%)	48/49 (98.0%)	48/49 (98.0%)	34/36 (94.4%)	545/567 (96.1%)
	**Control**	46/53 (86.8%)	51/54 (94.4%)	53/55 (96.4%)	58/62 (93.5%)	55/55 (100.0%)	58/63 (92.1%)	59/61 (96.7%)	50/56 (89.3%)	45/51 (88.2%)	61/70 (87.1%)	64/67 (95.5%)	59/60 (98.3%)	51/55 (92.7%)	710/762 (93.2%)
		p=0.469	p=1.000	p=1.000	p=0.135	p=1.000	p=0.397	p=1.000	p=0.031	p=0.031	p=0.883	p=0.637	p=1.000	p=1.000	**p=0.021**
**Patients' and families' understanding of condition**	**APHN**	25/43 (58.1%)	40/52 (76.9%)	25/36 (69.4%)	33/46 (71.7%)	33/42 (78.6%)	35/43 (81.4%)	35/41 (85.4%)	39/46 (84.8%)	36/42 (85.7%)	38/42 (90.5%)	44/49 (89.8%)	45/49 (91.8%)	31/36 (86.1%)	459/567 (81.0%)
	**Control**	32/53 (60.4%)	37/54 (68.5%)	33/55 (60.0%)	51/62 (82.3%)	43/55 (78.2%)	53/63 (84.1%)	49/61 (80.3%)	53/56 (94.6%)	35/51 (68.6%)	57/70 (81.4%)	48/67 (71.6%)	53/60 (88.3%)	43/55 (78.2%)	587/762 (77.0%)
		p=0.824	p=0.332	p=0.359	p=0.194	p=0.963	p=0.713	p=0.513	p=0.179	p=0.054	p=0.196	p=0.017	p=0.751	p=0.343	**p=0.084**
	**APHN**	5.6/11 (50.9%)	6.12/11 (55.6%)	6.86/11 (62.4%)	7.46/11 (67.8%)	7.86/11 (71.5%)	8.35/11 (75.9%)	8.95/11 (81.4%)	8.61/11 (78.3%)	8.43/11 (76.6%)	8.26/11 (75.1%)	8.65/11 (78.6%)	8.8/11 (80.0%)	9.31/11 (84.6%)	7.92/11 (72.0%)

	**mean ± SD**	5.6 ± 1.2	6.1 ± 1.5	6.9 ± 1.7	7.5 ± 1.5	7.9 ± 1.3	8.3 ± 1.9	9.0 ± 1.9	8.6 ± 1.8	8.4 ± 2.1	8.3 ± 1.5	8.7 ± 1.9	8.8 ± 1.6	9.3 ± 1.1	7.9 ± 1.9
**Overall adherence to PU recommendations**	**Control**	5.91/11 (53.7%)	5.41/11 (49.2%)	5.53/11 (50.3%)	5.69/11 (51.7%)	6.51/11 (59.2%)	5.98/11 (54.4%)	6/11 (54.5%)	6.2/11 (56.4%)	6.1/11 (55.5%)	6.07/11 (55.2%)	6.36/11 (57.8%)	7.1/11 (64.5%)	6.22/11 (56.5%)	6.09/11 (55.4%)
	**mean ± SD**	5.9 ± 1.3	5.4 ± 1.5	5.5 ± 1.2	5.7 ± 1.8	6.5 ± 1.4	6.0 ± 2.1	6.0 ± 1.8	6.2 ± 1.6	6.1 ± 1.8	6.1 ± 1.7	6.4 ± 1.7	7.1 ± 1.7	6.2 ± 2.0	6.0 ± 1.7
		p=0.240	p=0.016	p=0.000	p=0.000	p=0.000	p=0.000	p=0.000	p=0.000	p=0.000	p=0.000	p=0.000	p=0.000	p=0.000	**p=0.000**
**Pressure ulcer outcome indicators**															
		**Baseline**	**Month 1**	**Month 2**	**Mont 3**	**Month 4**	**Month 5**	**Month 6**	**Month 7**	**Month 8**	**Month 9**	**Month 10**	**Month 11**	**Month 12**	**Overall**
	**APHN**	12/151 (7.9%)	10/125 (8.0%)	4/130 (3.1%)	7/134 (5.2%)	6/139 (4.3%)	10/141 (7.1%)	6/131 (4.6%)	6/138 (4.3%)	3/141 (2.1%)	12/145 (8.3%)	10/149 (6.7%)	10/147 (6.8%)	7/126 (5.6%)	103/1797 (5.7%)
**Prevalence of PU**	**Control**	14/160 (8.8%)	11/153 (7.2%)	10/153 (6.5%)	11/156 (7.1%)	13/146 (8.9%)	15/146 (10.3%)	11/141 (7.8%)	15/144 (10.4%)	15/139 (10.8%)	17/146 (11.6%)	15/153 (9.8%)	12/157 (7.6%)	10/151 (6.6%)	169/1945 (8.7%)
		p=0.798	p=0.799	p=0.181	p=0.520	p=0.121	p=0.339	p=0.273	p=0.052	p=0.003	p=0.338	p=0.330	p=0.777	p=0.713	**p=0.000**
	**APHN**	11/41 (26.8%)	7/47 (14.9%)	4/35 (11.4%)	6/44 (13.6%)	6/42 (14.3%)	8/37 (21.6%)	6/42 (14.3%)	5/46 (10.9%)	3/42 (7.1%)	10/38 (26.3%)	8/47 (17.0%)	7/46 (15.2%)	7/38 (18.4%)	88/545 (16.1%)
**Prevalence of PU among patients at risk**	**Control**	12/53 (22.6%)	11/55 (20.0%)	9/53 (17.0%)	11/61 (18.0%)	12/54 (22.2%)	13/57 (22.8%)	9/58 (15.5%)	12/52 (23.1%)	13/50 (26.0%)	14/69 (20.3%)	15/66 (22.7%)	12/60 (20.0%)	9/55 (16.4%)	152/743 (20.5%)
		p=0.640	p=0.500	p=0.472	p=0.546	p=0.323	p=0.893	p=0.865	p=0.111	p=0.017	p=0.475	p=0.458	p=0.525	p=0.796	**p=0.049**
	**APHN**	12/151 (7.9%)	22/276 (8.0%)	26/406 (6.4%)	33/540 (6.1%)	39/679 (5.7%)	49/820 (6.0%)	55/951 (5.8%)	61/1089 (5.6%)	64/1230 (5.2%)	76/1375 (5.5%)	86/1524 (5.6%)	96/1671 (5.7%)	103/1797 (5.7%)	103/1797 (5.7%)
**Incidence of PU**	**Control**	14/160 (8.8%)	25/313 (8.0%)	35/466 (7.5%)	46/622 (7.4%)	59/768 (7.7%)	74/914 (8.1%)	85/1055 (8.1%)	100/1199 (8.3%)	115/1338 (8.6%)	132/1484 (8.9%)	147/1637 (9.0%)	159/1794 (8.9%)	169/1945 (8.7%)	169/1945 (8.7%)
		p=0.798	p=0.994	p=0.522	p=0.385	p=0.143	p=0.339	p=0.045	p=0.010	p=0.001	p=0.001	p=0.000	p=0.000	p=0.000	**p=0.000**
	**APHN**	11/41 (26.8%)	18/88 (20.5%)	22/123 (17.9%)	28/167 (16.8%)	34/209 (16.3%)	42/246 (17.1%)	48/288 (16.7%)	53/334 (15.9%)	56/376 (14.9%)	66/414 (15.9%)	74/461 (16.1%)	81/507 (16.0%)	88/545 (16.1%)	88/545 (16.1%)

**Incidence of PU among patients at risk**	**Control**	12/53 (22.6%)	23/108 (21.3%)	32/161 (19.9%)	43/222 (19.4%)	55/276 (19.9%)	68/333 (20.4%)	77/391 (19.7%)	89/443 (20.1%)	102/493 (20.7%)	116/562 (20.6%)	131/628 (20.9%)	143/688 (20.8%)	152/743 (20.5%)	152/743 (20.5%)
		p=0.639	p=0.885	p=0.672	p=0.510	p=0.302	p=0.310	p=0.314	p=0.131	p=0.028	p=0.062	p=0.044	p=0.035	p=0.049	**p=0.049**
**Other skin lesions**	**APHN**	1/43 (2.3%)	3/52 (5.8%)	2/36 (5.6%)	2/46 (4.3%)	2/42 (4.8%)	0/43 (0.0%)	3/41 (7.3%)	5/46 (10.9%)	3/42 (7.1%)	0/42 (0.0%)	2/49 (4.1%)	3/49 (6.1%)	2/36 (5.6%)	28/567 (4.9%)
	**Control**	4/53 (7.5%)	2/54 (3.7%)	1/55 (1.8%)	2/62 (3.2%)	2/55 (3.6%)	2/63 (3.2%)	4/61 (6.6%)	6/56 (10.7%)	4/51 (7.8%)	3/70 (4.3%)	3/67 (4.5%)	1/60 (1.7%)	2/55 (3.6%)	36/762 (4.7%)
		p=0.376	p=0.675	p=0.560	p=1.000	p=1.000	p=0.513	p=1.000	p=1.000	p=1.000	p=0.291	p=1.000	p=0.324	p=0.647	**p=0.897**

PU: pressure ulcer; APHN: Advanced practice hospitalization nurse; SD: Standard deviation
