# Supplementary material for: Impact of advanced practice nurses in hospital units on compliance with clinical practice guidelines: a quasi-experimental study
Source: BMC Nurs. 2022 Nov 29;21:331. doi: 10.1186/s12912-022-01110-x (PMC9706842; doi:10.1186/s12912-022-01110-x)
Supplement: Supplementary file 4 — Additional file 4. [file 12912_2022_1110_MOESM4_ESM.pdf]

Additional file 4. Evolution of process and outcome indicators derived from the CPG for vascular access devices

Vascular access devices process indicators															
		**Baseline**	**Month 1**	**Month 2**	**Month 3**	**Month 4**	**Month 5**	**Month 6**	**Month 7**	**Month 8**	**Month 9**	**Month 10**	**Month 11**	**Month 12**	**Overall**
**Catheters inserted in the antecubital fossa**	**APHN**	15/101 (14.9%)	15/101 (14.9%)	20/89 (22.5%)	21/93 (22.6%)	27/97 (27.8%)	23/90 (25.6%)	19/109 (17.4%)	20/109 (18.3%)	20/110 (18.2%)	24/100 (24.0%)	22/91 (24.2%)	13/98 (13.3%)	22/101 (21.8%)	261/1289 (20.2%)
	**Control**	20/92 (21.7%)	27/121 (22.3%)	27/115 (23.5%)	17/101 (16.8%)	24/96 (25.0%)	21/105 (20.0%)	24/89 (27.0%)	29/111 (26.1%)	24/108 (22.2%)	22/100 (22.0%)	17/91 (18.7%)	30/108 (27.8%)	26/95 (27.4%)	308/1332 (23.1%)
		p=0.215	p=0.157	p=0.866	p=0.313	p=0.655	p=0.355	p=0.106	p=0.166	p=0.457	p=0.737	p=0.366	p=0.010	p=0.363	**p=0.074**
**Catheters inserted in the lower limbs**	**APHN**	1/102 (1.0%)	0/101 (0.0%)	0/89 (0.0%)	0/93 (0.0%)	0/97 (0.0%)	0/90 (0.0%)	0/109 (0.0%)	0/109 (0.0%)	0/110 (0.0%)	0/100 (0.0%)	0/91 (0.0%)	0/98 (0.0%)	0/101 (0.0%)	1/1290 (0.1%)
	**Control**	0/92 (0.0%)	0/121 (0.0%)	2/117 (1.7%)	1/102 (1.0%)	3/99 (3.0%)	0/105 (0.0%)	0/89 (0.0%)	2/113 (1.8%)	0/108 (0.0%)	0/100 (0.0%)	1/92 (1.1%)	0/108 (0.0%)	0/95 (0.0%)	9/1341 (0.7%)
		p=1.000	NA	p=0.507	p=1.000	p=0.246	NA	NA	p=0.498	NA	NA	p=1.000	NA	NA	**p=0.021**
**Record of catheters inserted in the antecubital fossa**	**APHN**	2/62 (3.2%)	1/63 (1.6%)	6/74 (8.1%)	7/73 (9.6%)	2/79 (2.5%)	5/78 (6.4%)	6/85 (7.1%)	9/96 (9.4%)	11/92 (12.0%)	9/84 (10.7%)	8/74 (10.8%)	7/74 (9.5%)	4/86 (4.7%)	77/1020 (7.5%)
	**Control**	1/34 (2.9%)	4/59 (6.8%)	8/58 (13.8%)	2/41 (4.9%)	4/42 (9.5%)	2/60 (3.3%)	4/39 (10.3%)	5/55 (9.1%)	2/56 (3.6%)	2/42 (4.8%)	2/31 (6.5%)	0/38 (0.0%)	1/31 (3.2%)	37/586 (6.3%)
		p=1.000	p=0.196	p=0.292	p=0.485	p=0.181	p=0.699	p=0.724	p=0.954	p=0.132	p=0.334	p=0.720	p=0.093	p=1.000	**p=0.354**
**Record of catheters inserted in the lower limbs**	**APHN**	0/62 (0.0%)	0/63 (0.0%)	0/74 (0.0%)	0/73 (0.0%)	0/79 (0.0%)	0/78 (0.0%)	0/85 (0.0%)	0/96 (0.0%)	0/92 (0.0%)	0/84 (0.0%)	0/74 (0.0%)	0/74 (0.0%)	0/86 (0.0%)	0/1020 (0.0%)
	**Control**	0/34 (0.0%)	0/59 (0.0%)	2/60 (3.3%)	1/42 (2.4%)	2/44 (4.5%)	0/60 (0.0%)	0/39 (0.0%)	1/56 (1.8%)	0/56 (0.0%)	0/42 (0.0%)	1/32 (3.1%)	0/38 (0.0%)	0/31 (0.0%)	7/593 (1.2%)
		NA	NA	p=0.199	p=0.365	p=0.126	NA	NA	p=0.368	NA	NA	p=0.302	NA	NA	**p=0.001**
	**APHN**	45/62 (72.6%)	45/63 (71.4%)	62/74 (83.8%)	50/73 (68.5%)	64/79 (81.0%)	68/78 (87.2%)	73/85 (85.9%)	82/96 (85.4%)	86/92 (93.5%)	73/84 (86.9%)	64/74 (86.5%)	63/74 (85.1%)	77/86 (89.5%)	852/1020 (83.5%)

**Record of catheters inserted,with the orifice visible**	**Control**	18/34 (52.9%)	42/59 (71.2%)	45/60 (75.0%)	29/42 (69.0%)	31/44 (70.5%)	39/60 (65.0%)	31/39 (79.5%)	37/56 (66.1%)	33/56 (58.9%)	28/42 (66.7%)	24/32 (75.0%)	22/38 (57.9%)	22/31 (71.0%)	401/593 (67.6%)
		p=0.053	p=0.976	p=0.207	p=0.951	p=0.181	p=0.002	p=0.369	p=0.005	p=0.000	p=0.007	p=0.148	p=0.001	p=0.021	**p=0.000**
**Catheters inserted in a recommended location**	**APHN**	86/102 (84.3%)	86/101 (85.1%)	69/89 (77.5%)	72/93 (77.4%)	70/97 (72.2%)	67/90 (74.4%)	90/109 (82.6%)	89/109 (81.7%)	90/110 (81.8%)	76/100 (76.0%)	69/91 (75.8%)	85/98 (86.7%)	79/101 (78.2%)	1028/1290 (79.7%)
	**Control**	72/92 (78.3%)	94/121 (77.7%)	88/117 (75.2%)	84/102 (82.4%)	72/99 (72.7%)	84/105 (80.0%)	65/89 (73.0%)	82/113 (72.6%)	84/108 (77.8%)	78/100 (78.0%)	74/92 (80.4%)	78/108 (72.2%)	69/95 (72.6%)	1024/1341 (76.4%)
		p=0.279	p=0.157	p=0.699	p=0.390	p=0.930	p=0.355	p=0.106	p=0.108	p=0.457	p=0.737	p=0.451	p=0.010	p=0.363	**p=0.039**
**Catheters inserted, with the orifice visible**	**APHN**	68/102 (66.7%)	69/101 (68.3%)	73/89 (82.0%)	66/93 (71.0%)	77/97 (79.4%)	80/90 (88.9%)	88/109 (80.7%)	92/109 (84.4%)	103/110 (93.6%)	85/100 (85.0%)	76/91 (83.5%)	82/98 (83.7%)	91/101 (90.1%)	1050/1290 (81.4%)
	**Control**	53/92 (57.6%)	85/121 (70.2%)	77/117 (65.8%)	66/102 (64.7%)	66/99 (66.7%)	70/105 (66.7%)	63/89 (70.8%)	74/113 (65.5%)	62/108 (57.4%)	62/100 (62.0%)	63/92 (68.5%)	69/108 (63.9%)	67/95 (70.5%)	877/1341 (65.4%)
		p=0.193	p=0.756	p=0.010	p=0.350	p=0.045	p=0.000	p=0.102	p=0.001	p=0.000	p=0.000	p=0.017	p=0.001	p=0.001	**p=0.000**
**Catheters in use**	**APHN**	87/102 (85.3%)	99/101 (98.0%)	85/89 (95.5%)	85/93 (91.4%)	87/97 (89.7%)	76/90 (84.4%)	99/109 (90.8%)	98/109 (89.9%)	97/110 (88.2%)	88/100 (88.0%)	79/91 (86.8%)	80/98 (81.6%)	90/101 (89.1%)	1150/1290 (89.1%)
	**Control**	85/92 (92.4%)	117/121 (96.7%)	100/117 (85.5%)	81/102 (79.4%)	86/99 (86.9%)	80/105 (76.2%)	78/89 (87.6%)	90/113 (79.6%)	83/108 (76.9%)	86/100 (86.0%)	80/92 (87.0%)	84/108 (77.8%)	79/95 (83.2%)	1129/1341 (84.2%)
		p=0.120	p=0.691	p=0.018	p=0.019	p=0.539	p=0.151	p=0.469	p=0.034	p=0.027	p=0.674	p=0.977	p=0.493	p=0.227	**p=0.000**
**Duration of catheter insertion**	**APHN**	67/102 (65.7%)	68/101 (67.3%)	64/89 (71.9%)	57/93 (61.3%)	62/97 (63.9%)	52/90 (57.8%)	64/109 (58.7%)	64/109 (58.7%)	74/110 (67.3%)	55/100 (55.0%)	61/91 (67.0%)	63/98 (64.3%)	61/101 (60.4%)	812/1290 (62.9%)
	**Control**	49/92 (53.3%)	52/121 (43.0%)	70/117 (59.8%)	40/102 (39.2%)	49/99 (49.5%)	55/105 (52.4%)	36/89 (40.4%)	49/113 (43.4%)	51/108 (47.2%)	41/100 (41.0%)	38/92 (41.3%)	44/108 (40.7%)	45/95 (47.4%)	619/1341 (46.2%)
		p=0.078	p=0.000	p=0.072	p=0.002	p=0.042	p=0.450	p=0.011	p=0.022	p=0.003	p=0.048	p=0.000	p=0.001	p=0.067	**p=0.000**
**Type of catheter attachment**	**APHN**	68/102 (66.7%)	69/101 (68.3%)	71/89 (79.8%)	65/93 (69.9%)	77/97 (79.4%)	79/90 (87.8%)	87/109 (79.8%)	90/109 (82.6%)	102/110 (92.7%)	83/100 (83.0%)	76/91 (83.5%)	81/98 (82.7%)	89/101 (88.1%)	1037/1290 (80.4%)
	**Control**	52/92 (56.5%)	85/121 (70.2%)	76/117 (65.0%)	66/102 (64.7%)	65/99 (65.7%)	67/105 (63.8%)	60/89 (67.4%)	72/113 (63.7%)	59/108 (54.6%)	62/100 (62.0%)	63/92 (68.5%)	68/108 (63.0%)	66/95 (69.5%)	861/1341 (64.2%)
		p=0.146	p=0.756	p=0.020	p=0.441	p=0.032	p=0.000	p=0.047	p=0.002	p=0.000	p=0.001	p=0.017	p=0.002	p=0.001	**p=0.000**

**Condition of the dressing**	**APHN**	61/102 (59.8%)	69/101 (68.3%)	63/89 (70.8%)	63/93 (67.7%)	71/97 (73.2%)	73/90 (81.1%)	88/109 (80.7%)	68/109 (62.4%)	67/110 (60.9%)	72/100 (72.0%)	67/91 (73.6%)	85/98 (86.7%)	76/101 (75.2%)	923/1290 (71.6%)
	**Control**	54/92 (58.7%)	65/121 (53.7%)	54/117 (46.2%)	47/102 (46.1%)	55/99 (55.6%)	66/105 (62.9%)	54/89 (60.7%)	66/113 (58.4%)	57/108 (52.8%)	57/100 (57.0%)	52/92 (56.5%)	72/108 (66.7%)	64/95 (67.4%)	763/1341 (56.9%)
		p=0.875	p=0.027	p=0.000	p=0.002	p=0.010	p=0.005	p=0.002	p=0.545	p=0.225	p=0.027	p=0.015	p=0.001	p=0.222	**p=0.000**
**Catheter record**	**APHN**	62/102 (60.8%)	63/101 (62.4%)	74/89 (83.1%)	73/93 (78.5%)	79/97 (81.4%)	78/90 (86.7%)	85/109 (78.0%)	96/109 (88.1%)	92/110 (83.6%)	84/100 (84.0%)	74/91 (81.3%)	74/98 (75.5%)	86/101 (85.1%)	1020/1290 (79.1%)
	**Control**	34/92 (37.0%)	59/121 (48.8%)	60/117 (51.3%)	42/102 (41.2%)	44/99 (44.4%)	60/105 (57.1%)	39/89 (43.8%)	56/113 (49.6%)	56/108 (51.9%)	42/100 (42.0%)	32/92 (34.8%)	38/108 (35.2%)	31/95 (32.6%)	593/1341 (44.2%)
		p=0.001	p=0.042	p=0.000	p=0.000	p=0.000	p=0.000	p=0.000	p=0.000	p=0.000	p=0.000	p=0.000	p=0.000	p=0.000	**p=0.000**
**Overall Adherence to VA recommendations**	**APHN**	4.89/7 (69.9%)	5.18/7 (74.0%)	5.61/7 (80.1%)	5.17/7 (73.9%)	5.39/7 (77.0%)	5.61/7 (80.1%)	5.51/7 (78.7%)	5.48/7 (78.3%)	5.68/7 (81.1%)	5.43/7 (77.6%)	5.52/7 (78.9%)	5.61/7 (80.1%)	5.66/7 (80.9%)	5.44/7 (77.7%)
	**mean ± SD**	4.9 ± 1.5	5.2 ± 1.5	5.6 ± 1.5	5.2 ± 1.5	5.4 ± 1.4	5.6 ± 1.3	5.5 ± 1.4	5.5 ± 1.4	5.7 ± 1.2	5.4 ± 1.2	5.5 ± 1.3	5.6 ± 1.2	5.7 ± 1.3	5.4 ± 1.4
	**Control**	4.34/7 (62.0%)	4.6/7 (65.7%)	4.49/7 (64.1%)	4.18/7 (59.7%)	4.41/7 (63.0%)	4.59/7 (65.6%)	4.44/7 (63.4%)	4.33/7 (61.9%)	4.19/7 (59.9%)	4.28/7 (61.1%)	4.37/7 (62.4%)	4.19/7 (59.9%)	4.43/7 (63.3%)	4.37/7 (62.4%)
	**mean ± SD**	4.3 ± 1.5	4.6 ± 1.6	4.5 ± 1.6	4.2 ± 1.5	4.4 ± 1.7	4.6 ± 1.5	4.4 ± 1.6	4.3 ± 1.7	4.2 ± 1.6	4.3 ± 1.6	4.4 ± 1.4	4.2 ± 1.5	4.4 ± 1.6	4.4 ± 1.6
		p=0.009	p=0.006	p=0.000	p=0.000	p=0.000	p=0.000	p=0.000	p=0.000	p=0.000	p=0.000	p=0.000	p=0.000	p=0.000	**p=0.000**
**Vascular access devices outcome indicators**															
		**Baseline**	**Month 1**	**Month 2**	**Month 3**	**Month 4**	**Month 5**	**Month 6**	**Month 7**	**Month 8**	**Month 9**	**Month 10**	**Month 11**	**Month 12**	**Overall**
**Prevalence of adverse events**	**APHN**	25/102 (24.5%)	21/101 (20.8%)	12/89 (13.5%)	13/93 (14.0%)	13/97 (13.4%)	11/90 (12.2%)	15/109 (13.8%)	16/109 (14.7%)	12/110 (10.9%)	9/100 (9.0%)	14/91 (15.4%)	10/98 (10.2%)	9/101 (8.9%)	180/1290 (14.0%)
	**Control**	31/92 (33.7%)	36/121 (29.8%)	22/117 (18.8%)	27/102 (26.5%)	24/99 (24.2%)	18/105 (17.1%)	11/89 (12.4%)	17/113 (15.0%)	26/108 (24.1%)	20/100 (20.0%)	19/92 (20.7%)	19/108 (17.6%)	19/95 (20.0%)	289/1341 (21.6%)
		p=0.159	p=0.128	p=0.308	p=0.031	p=0.053	p=0.336	p=0.771	p=0.939	p=0.010	p=0.027	p=0.354	p=0.128	p=0.027	**p=0.000**
**Incidence of adverse events**	**APHN**	25/102 (24.5%)	46/203 (22.7%)	58/292 (19.9%)	71/385 (18.4%)	84/482 (17.4%)	95/572 (16.6%)	110/681 (16.2%)	126/790 (15.9%)	138/900 (15.3%)	147/1000 (14.7%)	161/1091 (14.8%)	171/1189 (14.4%)	180/1290 (14.0%)	180/1290 (14.0%)

	**Control**	31/92 (33.7%)	67/213 (31.5%)	89/330 (27.0%)	116/432 (26.9%)	140/531 (26.4%)	158/636 (24.8%)	169/725 (23.3%)	186/838 (22.2%)	212/946 (22.4%)	232/1046 (22.2%)	251/1138 (22.1%)	270/1246 (21.7%)	289/1341 (21.6%)	289/1341 (21.6%)
		p=0.158	p=0.043	p=0.037	p=0.004	p=0.000	p=0.000	p=0.000	p=0.001	p=0.000	p=0.000	p=0.000	p=0.000	p=0.000	**p=0.000**
**Erythema**	**APHN**	8/96 (8.3%)	1/99 (1.0%)	5/87 (5.7%)	2/89 (2.2%)	3/93 (3.2%)	4/89 (4.5%)	3/107 (2.8%)	4/107 (3.7%)	1/109 (0.9%)	2/100 (2.0%)	3/91 (3.3%)	1/97 (1.0%)	1/99 (1.0%)	38/1263 (3.0%)
	**Control**	13/87 (14.9%)	10/117 (8.5%)	4/108 (3.7%)	2/94 (2.1%)	2/91 (2.2%)	5/97 (5.2%)	2/85 (2.4%)	4/105 (3.8%)	5/98 (5.1%)	7/90 (7.8%)	6/88 (6.8%)	8/107 (7.5%)	2/93 (2.2%)	70/1260 (5.6%)
		p=0.161	p=0.012	p=0.516	p=1.000	p=1.000	p=1.000	p=1.000	p=1.000	p=0.103	p=0.087	p=0.324	p=0.037	p=0.612	**p=0.002**
**Venous cord**	**APHN**	1/96 (1.0%)	0/99 (0.0%)	0/87 (0.0%)	0/89 (0.0%)	0/93 (0.0%)	0/89 (0.0%)	1/107 (0.9%)	1/107 (0.9%)	0/109 (0.0%)	0/100 (0.0%)	0/91 (0.0%)	0/97 (0.0%)	1/99 (1.0%)	4/1263 (0.3%)
	**Control**	3/87 (3.4%)	3/117 (2.6%)	1/108 (0.9%)	1/94 (1.1%)	1/91 (1.1%)	0/97 (0.0%)	0/85 (0.0%)	1/105 (1.0%)	5/98 (5.1%)	1/90 (1.1%)	0/88 (0.0%)	3/107 (2.8%)	4/93 (4.3%)	23/1260 (1.8%)
		p=0.348	p=0.252	p=1.000	p=1.000	p=0.495	NA	p=1.000	p=1.000	p=0.023	p=0.474	NA	p=0.248	p=0.200	**p=0.000**
**Inflammation**	**APHN**	3/96 (3.1%)	2/99 (2.0%)	0/87 (0.0%)	0/89 (0.0%)	1/93 (1.1%)	2/89 (2.2%)	2/107 (1.9%)	1/107 (0.9%)	2/109 (1.8%)	4/100 (4.0%)	1/91 (1.1%)	1/97 (1.0%)	2/99 (2.0%)	21/1263 (1.7%)
	**Control**	2/87 (2.3%)	3/117 (2.6%)	1/108 (0.9%)	2/94 (2.1%)	3/91 (3.3%)	2/97 (2.1%)	2/85 (2.4%)	1/105 (1.0%)	3/98 (3.1%)	1/90 (1.1%)	3/88 (3.4%)	2/107 (1.9%)	5/93 (5.4%)	30/1260 (2.4%)
		p=1.000	p=1.000	p=1.000	p=0.498	p=0.365	p=1.000	p=1.000	p=1.000	p=0.669	p=0.372	p=0.362	p=1.000	p=0.267	**p=0.200**
**Pain**	**APHN**	16/102 (15.7%)	16/101 (15.8%)	11/89 (12.4%)	5/93 (5.4%)	9/97 (9.3%)	7/90 (7.8%)	6/109 (5.5%)	11/109 (10.1%)	10/110 (9.1%)	6/100 (6.0%)	7/91 (7.7%)	7/98 (7.1%)	7/101 (6.9%)	118/1290 (9.1%)
	**Control**	11/92 (12.0%)	13/121 (10.7%)	7/117 (6.0%)	13/102 (12.7%)	14/99 (14.1%)	7/105 (6.7%)	4/89 (4.5%)	8/113 (7.1%)	10/108 (9.3%)	7/100 (7.0%)	5/92 (5.4%)	8/108 (7.4%)	13/95 (13.7%)	120/1341 (8.9%)
		p=0.454	p=0.262	p=0.108	p=0.076	p=0.290	p=0.764	p=0.747	p=0.423	p=0.966	p=0.774	p=0.537	p=0.942	p=0.119	**p=0.859**
**Unclassified adverse event**	**APHN**	6/96 (6.3%)	4/99 (4.0%)	1/87 (1.1%)	7/89 (7.9%)	1/93 (1.1%)	1/89 (1.1%)	7/107 (6.5%)	3/107 (2.8%)	/109 (0.0%)	/100 (0.0%)	3/91 (3.3%)	1/97 (1.0%)	1/99 (1.0%)	35/1263 (2.8%)
	**Control**	9/87 (10.3%)	14/117 (12.0%)	10/108 (9.3%)	12/94 (12.8%)	9/91 (9.9%)	6/97 (6.2%)	5/85 (5.9%)	6/105 (5.7%)	8/98 (8.2%)	5/90 (5.6%)	7/88 (8.0%)	3/107 (2.8%)	1/93 (1.1%)	95/1260 (7.5%)
		p=0.313	p=0.036	p=0.024	p=0.277	p=0.009	p=0.120	p=0.851	p=0.330	p=0.002	p=0.022	p=0.207	p=0.623	p=1.000	**p=0.005**

**Unnecessary catheters**	**APHN**	15/102 (14.7%)	2/101 (2.0%)	4/89 (4.5%)	8/93 (8.6%)	10/97 (10.3%)	14/90 (15.6%)	10/109 (9.2%)	11/109 (10.1%)	13/110 (11.8%)	12/100 (12.0%)	12/91 (13.2%)	18/98 (18.4%)	11/101 (10.9%)	140/1290 (10.9%)
	**Control**	7/92 (7.6%)	4/121 (3.3%)	17/117 (14.5%)	21/102 (20.6%)	13/99 (13.1%)	25/105 (23.8%)	11/89 (12.4%)	23/113 (20.4%)	25/108 (23.1%)	14/100 (14.0%)	12/92 (13.0%)	24/108 (22.2%)	16/95 (16.8%)	212/1341 (15.8%)
		p=0.120	p=0.691	p=0.018	p=0.019	p=0.539	p=0.151	p=0.469	p=0.034	p=0.027	p=0.674	p=0.977	p=0.493	p=0.227	**p=0.000**

VA: Vascular access; NA: Not applicable
